# Supplementary material for: Relative Risks of Adverse Perinatal Outcomes in Three Australian Communities Exposed to Per- and Polyfluoroalkyl Substances: Data Linkage Study
Source: Int J Environ Res Public Health. 2023 Oct 5;20(19):6886. doi: 10.3390/ijerph20196886 (PMC10572760; doi:10.3390/ijerph20196886)
Supplement: Supplementary file 1 [file ijerph-20-06886-s001.zip › ijerph-2512793-supplementary.pdf]

Supplementary Table S1. Comparison of perinatal outcomes in exposed and comparison populations: adjusted relative risks (RR) of adverse perinatal outcomes, and adjusted difference in means of growth measurements, where continuous covariates were modelled as categorical variables, and where gestational diabetes was included as an additional covariate

	NT			Qld			NSW		
	**Adjusted RR[1] (95% CI)**	**Adjusted RR[2] (95% CI)**	**Adjusted RR[3] (95% CI)**	**Adjusted RR[1] (95% CI)**	**Adjusted RR[2] (95% CI)**	**Adjusted RR[3] (95% CI)**	**Adjusted RR[1] (95% CI)**	**Adjusted RR[2] (95% CI)**	**Adjusted RR[3] (95% CI)**
Adverse perinatal outcome									
Gestational diabetes	1.07 (0.91,1.27)	1.10 (0.93,1.30)		0.95 (0.71,1.26)	0.93 (0.69,1.25)		1.45 (0.86,2.46)	1.43 (0.85,2.41)	
Pregnancy-induced hypertension	0.92 (0.80,1.07)	1.11 (0.93,1.33)		1.02 (0.61,1.71)	0.99 (0.59,1.67)		1.98 (1.35,2.90)	1.88 (1.29,2.73)	
Caesarean/assisted vaginal	0.99 (0.94,1.04)	0.96 (0.91,1.02)	0.98 (0.92,1.04)	1.13 (1.01,1.26)	1.13 (1.01,1.27)	1.12 (1.00,1.26)	1.03 (0.84,1.27)	0.99 (0.82,1.21)	0.92 (0.74,1.14)
Emergency caesarean	0.99 (0.90,1.09)	1.07 (0.96,1.20)		1.10 (0.89,1.38)	1.14 (0.91,1.42)		1.23 (0.83,1.81)	1.13 (0.77,1.67)	
Postpartum haemorrhage	0.95 (0.85,1.06)	1.01 (0.90,1.13)		0.96 (0.70,1.32)	0.90 (0.63,1.27)		1.89 (1.09,3.28)	1.87 (1.07,3.26)	
Preterm birth	0.95 (0.85,1.07)	1.06 (0.92,1.22)		1.12 (0.85,1.48)	1.05 (0.77,1.42)		1.47 (0.89,2.41)	1.48 (0.90,2.43)	

Spontaneous preterm birth	0.99 (0.86,1.14)	1.14 (0.96,1.36)		0.96 (0.65,1.44)	0.88 (0.57,1.36)		1.28 (0.61,2.70)	1.32 (0.63,2.79)	
Small for gestational age	0.87 (0.80,0.95)	0.92 (0.82,1.03)		0.94 (0.64,1.39)	1.01 (0.68,1.50)		0.88 (0.49,1.58)	0.87 (0.49,1.54)	
Large for gestational age	0.96 (0.86,1.08)	0.92 (0.80,1.04)	0.86 (0.74,0.99)	0.93 (0.66,1.31)	0.91 (0.64,1.30)	0.89 (0.62,1.27)	0.90 (0.58,1.38)	0.96 (0.63,1.46)	1.06 (0.69,1.63)
Stillbirth	0.95 (0.66,1.36)	0.90 (0.54,1.51)		2.42 (1.19,4.94)	2.59 (1.25,5.39)		1.22 (0.16,9.02)	1.29 (0.17,9.54)	
Low Apgar at 5 min	0.94 (0.78,1.13)	1.06 (0.83,1.36)		1.50 (0.99,2.27)	1.48 (0.96,2.29)		1.01 (0.38,2.65)	0.98 (0.37,2.60)	
**Term (≥37 weeks) outcome**									
Term low Apgar score at 5 min	0.78 (0.60,1.01)	0.84 (0.59,1.20)		1.16 (0.66,2.02)	1.20 (0.69,2.10)		1.61 (0.60,4.32)	1.56 (0.58,4.22)	

	**NT**			**Qld**			**NSW**		
	**Adjusted difference in means[1] (95% CI)**	**Adjusted difference in means[2] (95% CI)**	**Adjusted difference in means[3] (95% CI)**	**Adjusted difference in means[1] (95% CI)**	**Adjusted difference in means[2] (95% CI)**	**Adjusted difference in means[3] (95% CI)**	**Adjusted difference in means[1] (95% CI)**	**Adjusted difference in means[2] (95% CI)**	**Adjusted difference in means[3] (95% CI)**
Growth measure									
Term birth weight (g)	30.7 (14.0,47.4)	10.4 (−9.7,30.4)	−0.7 (−22.7,21.4)	−10.8 (−50.9,29.3)	−7.4 (−45.0,30.2)	−11.3 (−48.9,26.3)	22.2 (−48.0,92.4)	35.6 (−29.5,100.8)	52.3 (−19.1,123.7)
Term birth length (cm)	0.3 (0.1,0.4)	0.2 (0.1,0.4)		0.3 (0.1,0.5)	0.3 (0.1,0.6)				

Term head circumference (cm)	0.1 (0.0,0.2)	0.0 (−0.1,0.1)		0.0 (−0.1,0.2)	0.0 (−0.1,0.2)

Table notes

The RR is the risk in the exposed group divided by the risk in the comparison group. The difference in means is the mean in the exposed group minus the mean in the comparison group.

1. RRs/Difference in means from Model 1: adjusted for year of birth, maternal age and mother’ s Aboriginal and Torres Strait Islander status (except NSW). Outcomes restricted to term babies included adjustment for gestational week. Year of birth and maternal age were treated as categorical covariates.
2. RRs/Difference in means from Model 2: adjusted for year of birth, maternal age, maternal Aboriginal and Torres Strait Islander status (except NSW), parity, marital status (except NSW), maternal country of birth, maternal BMI (Qld only) and maternal ever smoked during pregnancy. Caesarean/assisted vaginal, emergency caesarean and postpartum haemorrhage were additionally adjusted for macrosomia. Preterm birth, still birth, low Apgar and growth measures were additionally adjusted for sex of baby. Outcomes restricted to term babies included adjustment for gestational week. Year of birth, maternal age and maternal BMI were treated as categorical covariates.
3. RR/Mean difference adjusted for year of birth, maternal age, maternal Aboriginal and Torres Strait Islander status (except NSW), parity, marital status (except NSW), maternal country of birth, maternal BMI (Qld only) maternal ever smoke during pregnancy, and gestational diabetes. Caesarean/assisted vaginal were additionally adjusted for macrosomia. The analysis of term birthweight included adjustment for gestational week.

Supplementary Table S2. Comparison of perinatal outcomes in exposed and comparison populations: risks (%) adjusted relative risks (RR) of a composite adverse infant outcome.

	NT				Qld				NSW			
	**Exposed % (n)**	**Comparison % (n)**	**Adjusted RR¹ (95% CI)**	**Adjusted RR² (95% CI)**	**Exposed % (n)**	**Comparison % (n)**	**Adjusted RR¹ (95% CI)**	**Adjusted RR² (95% CI)**	**Exposed % (n)**	**Comparison % (n)**	**Adjusted RR¹ (95% CI)**	**Adjusted RR² (95% CI)**
**Total sample**	**5,606**	**11,364**			**665**	**3,989**			**188**	**7,287**		
Composite adverse infant outcome	29% (1,628)	30% (3,392)	0.93 (0.88,0.98)	0.96 (0.90,1.02)	19% (127)	19% (772)	0.96 (0.81,1.15)	0.98 (0.82,1.17)	26% (49)	25% (1,820)	1.04 (0.81,1.33)	1.05 (0.82,1.34)

Table notes

The RR is the risk in the exposed group divided the risk in comparison group. Composite outcome comprised any of the following events: preterm birth, small for gestational age, large for gestational age, still birth and low Apgar score.

1. RRs from Model 1: adjusted for year of birth, maternal age and mother' s Aboriginal and Torres Strait Islander status (except NSW).
2. RRs from Model 2: adjusted for year of birth, maternal age, maternal Aboriginal and Torres Strait Islander status (except NSW), parity, marital status (except NSW), maternal country of birth, maternal BMI (Qld only), maternal ever smoked during pregnancy and sex of baby.
